# Supplementary material for: Reconstruction of evolving nanostructures in ultrathin films with X-ray waveguide fluorescence holography
Source: Nat Commun. 2020 Jun 24;11:3197. doi: 10.1038/s41467-020-16980-5 (PMC7314812; doi:10.1038/s41467-020-16980-5)
Supplement: Supplementary file 1 — Supplementary Information [file 41467_2020_16980_MOESM1_ESM.pdf]

Supplementary Information for

Reconstruction of Evolving Nanostructures in Ultrathin Films with X-ray Waveguide Fluorescence Holography

Zhang Jiang et al.

Supplementary Note 1: Cubic b-spline as basis functions for arbitrary profiles

A cubic b-spline is a collection of piecewise third-order polynomials jointed together to approximate a smooth curve[1]. As basis functions, they can produce a curvature whose second derivative is continuous. Supplementary Figure 1 illustrates how cubic b-spline basis functions are used to synthesize arbitrary curves.

Supplementary Note 2: A brief introduction to Hamiltonian Markov Chain Monte Carlo (HMCMC) and its application to XWFH reconstruction

Assume the probability of the measured intensity is a normal distribution such that $I_{\mathrm{F},j}^{\mathrm{mea}} \sim \mathcal{N}\left(I_{\mathrm{F},j}^{\mathrm{cal}}, \sigma^2\right)$ for the jth $(j =, \cdots, M)$ data point. If we have no prior knowledge about the unknown parameters $\boldsymbol{x} = \{x_1, \dots, x_N\}$, the conditional posterior joint-distribution of $\boldsymbol{x}$ set is given by Bayes' theory

$$P(\boldsymbol{x}|I_{\mathrm{F}}^{\mathrm{mea}}) \propto P(I_{\mathrm{F}}^{\mathrm{mea}}|\boldsymbol{x}) = \prod_{j=1}^{M} \frac{1}{\sqrt{2\pi\sigma^2}} \exp\left[-\frac{\left(I_{\mathrm{F},j}^{\mathrm{mea}} - I_{\mathrm{F},j}^{\mathrm{cal}}\right)^2}{2\sigma^2}\right] \tag{1}$$

Knowing this target distribution, one can evaluate the statistics of the parameters, as well as the statistics of variables or functions that are derived from these parameters, such as mean, mode, variance, etc. However, this target distribution is not easily obtained in analytical forms, especially for highly nonlinear and high-dimensional models such as in many inversion problems in the field of X-ray and neutron scattering, imaging or spectroscopy. Therefore, Markov Chain Monte Carlo (MCMC) methods become popular recently[2], and they draw a large number of samples from this distribution to numerically represent the joint distribution of the parameters and the probability distribution of derived variables. Another advantage of the MCMC is that the correlation of the parameters is obtained naturally and the impact of their joint distributions on the probability or

uncertainty analysis of the derived functions, for example, the gold density profile, can be evaluated, which is not readily possible with conventional cost-minimization based optimization methods such as least-square fitting. Although classical random-walk based MCMC samples are often used, the sampling efficiency for a high-dimension parameter space is very low because MCMC is practically unable to explore the entire space (this is known as the curse of dimensionality). In addition, the effective number of independent samples is small due to the large serial correlation along the sequence. Hamiltonian MCMC is a recently developed MCMC variation to overcome these problems[3,4]. It makes an analog to the Hamiltonian dynamics in classical mechanics. Briefly, $\boldsymbol{x}$ is viewed as the generalized coordinate vector in the parameter space, and the potential energy is defined as

$$U(\boldsymbol{x}) = -\log P(\boldsymbol{x}) \tag{2}$$

For simplicity, we dropped the conditioning on $I_{\mathrm{F}}^{\mathrm{mea}}$. Notice that $U(\boldsymbol{x})$ is equivalent to the SSR in the cost function J in the main text. Adding the regularization terms, we have[5]

$$U(\boldsymbol{x}) = \frac{1}{2\sigma^2}\sum_{j=1}^{M}\left(I_{\mathrm{F},j}^{\mathrm{mea}} - I_{\mathrm{F},j}^{\mathrm{cal}}\right)^2 + M\log\sqrt{2\pi\sigma^2} + \frac{1}{2\sigma^2}\left(\beta_1\sum_{i=1}^{N}|C_i| + \beta_2\sum_{i=1}^{N-1}|\Delta C_i|^2\right) \tag{3}$$

Here $\boldsymbol{x}$ is the collection of all unknown parameters to be optimized, and it also includes the unknown variance σ^2 of the residuals.

Generalized momentum vector $\boldsymbol{p} = \{p_1, \dots, p_N\}$ is introduced as an auxiliary variable. The kinetic energy of the system is $K(\boldsymbol{p}) = -\log P(\boldsymbol{p})$. The probability distribution of $\boldsymbol{p}$ often assumes a multivariate normal distribution with an identity covariance, such that $P(\boldsymbol{p}) \propto \exp\left(-\frac{1}{2}|\boldsymbol{p}|^2\right)$. The joint posterior distribution of $\{\boldsymbol{x},\boldsymbol{p}\}$ for drawing a new sample is given by

$$P(\boldsymbol{x},\boldsymbol{p}) = P(\boldsymbol{x})P(\boldsymbol{p}) \propto \exp[-H(\boldsymbol{x},\boldsymbol{p})] \tag{4}$$

where $H(\boldsymbol{x},\boldsymbol{p}) = U(\boldsymbol{x}) + K(\boldsymbol{p})$ takes the form of the Hamiltonian in classical mechanics. It has been shown that this auxiliary momentum facilitates the HMCMC to more efficiently explore

further distances and a larger volume in the high-dimensional parameter space than conventional random-walk based MCMC, reduce the serial correlation of the sequence and lower the chance of being locally trapped.

The algorithm of a simple-version HMCMC works as follows. Given a parameter vector $\boldsymbol{x}_k$ at the kth step, a momentum vector $\boldsymbol{p}_k$ is drawn randomly to form a state of $(\boldsymbol{x}_k, \boldsymbol{p}_k)$ whose Hamiltonian is $H(\boldsymbol{x}_k, \boldsymbol{p}_k) = U(\boldsymbol{x}_k) + K(\boldsymbol{p}_k)$. The evolution to the next state $(\boldsymbol{x}_{k+1}, \boldsymbol{p}_{k+1})$ is achieved through a frictionless motion depicted by the Hamiltonian equation

$$\frac{\partial \boldsymbol{x}}{\partial t} = \frac{\partial H}{\partial \boldsymbol{p}} = \boldsymbol{p} \tag{5}$$

$$\frac{\partial \boldsymbol{p}}{\partial t} = -\frac{\partial H}{\partial \boldsymbol{x}} = -\frac{\partial U(\boldsymbol{x})}{\partial \boldsymbol{x}} \tag{6}$$

In practice, this evolution is numerically performed with the leapfrog integrator[3]. We then discard $\boldsymbol{p}_{k+1}$ and replace it with another randomly drawn momentum vector for HMCMC to continue the next iteration. The property of the frictionless Hamiltonian dynamics automatically guarantees the detailed balance of MCMC, because reversing the direction of the momentum vector time-reverses the evolution of the system. In theory, the acceptance rate is 100% (due to the conservation of energy), giving rise to the high efficiency of HMCMC. However, due to the numerical error of the leapfrogging, the Hamiltonian is not precisely a constant, and the new state is accepted with probability $\min\{1, \exp[-H(\boldsymbol{x}_{k+1}, \boldsymbol{p}_{k+1}) + H(\boldsymbol{x}_k, \boldsymbol{p}_k)]\}$.

With properly chosen step size and evolution lengths, HMCMC can converge quickly, even with the weakest guess in the current study, i.e., the gold number distribution is uniform throughout the entire film. It took only several iterations for the HMCMC to converge, as shown in Supplementary Figure 2 and 3. The sampling is stable after convergence, implying a unique solution. We collected 1000 stationary samplings and used them to calculate statistics of the gold atomic number distributions, such as mean, mode, median as well as the confidence. The very

narrow uncertainty band of the gold profile in Supplementray Figure 4 suggests that with our reconstruction algorithm, the XWFH is a high-precision tool for nanostructure characterization in thin films. Here, we adopt the conventions in statistics and define the uncertainty of a parameter or a function at the 95% confidence level (between the 2.5% and 97.5% percentiles of the probability distribution for the gold atom profile based on the many possible profiles calculated from HMCMC-sampled parameters). A caveat worthy of attention when interpreting the uncertainty is that it is obtained based on the given model used for the analysis so that this uncertainty only reflects the flexibility of the model and does not necessarily reflect the confidence with respect to the ground truth. According to the law of parsimony, the best practice is to accept a model that reasonably well (but not the best in terms of the χ^2 or the sum of the residual squares as adopted in the least-square minimizations) describes the measurement data and that has a physical meaning as well as the fewest number of parameters. For that purpose, cross-model assessment is required. To be specific, one needs to estimate parameters for all candidate models, and then compare them with model-selection criteria such as Akaike's information criterion (AIC), Schwarz's Bayesian information criterion (BIC), minimum description length (MDL), etc[1]. Here, we apply the simple mean-square-residual (MSR) criteria, as shown in the inset of Figs. 3c and 3h, to minimize the number of parameters for modeling the gold density profile. Therefore, in this work, the high confidence of the profile with XWFH also arises from the thorough within-model and cross-model analysis and this result is also experimentally validated by two independent techniques GISAXS and reflectivity.

Supplementary Note 3: Profile variance sensitivity comparison of GISAXS and XWFH

As shown in Figure 3 of the main article, GISAXS data has a tendency of slightly overestimating the width of the atom distribution profile. To illustrate this in detail, the σ value of the profile was varied in a ±10 Å range around the reconstructed values of GISAXS and XWFH. Corresponding mean square residuals (MSR) are calculated and displayed in Supplementray Figure 5. It is seen that given identical positive Δσ offset, XWFH gives a larger MSR, indicating a higher penalty for the XWFH cost function and hence a higher sensitivity of the XWFH than GISAXS.

Supplementary Figures

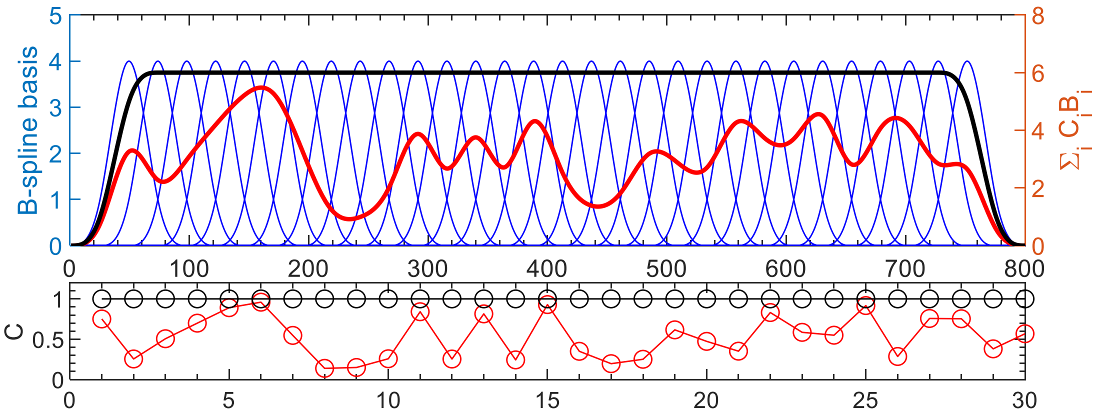

Supplementray Figure 1 Illustration of 30 cubic b-spline basis $\{B_{i=1,\cdots,30}\}$ with uniformly spaced knots. They are basis functions to produce arbitrary smooth curves (top panel) given a set of constant (blue) or random (red) coefficients $\{C_{i=1,\cdots,30}\}$ (bottom panel).

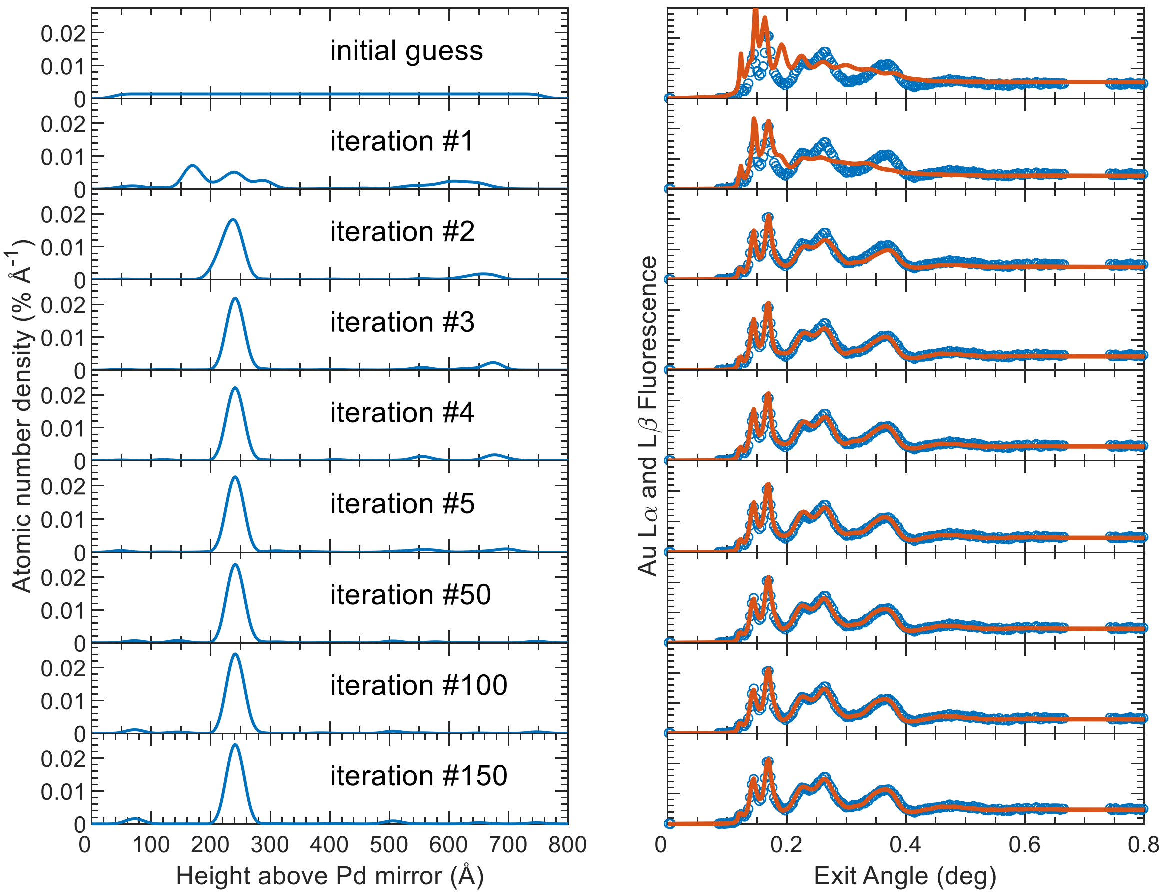

Supplementray Figure 2 Initial guess and selected HMCMC iterations for XWFH before annealing.

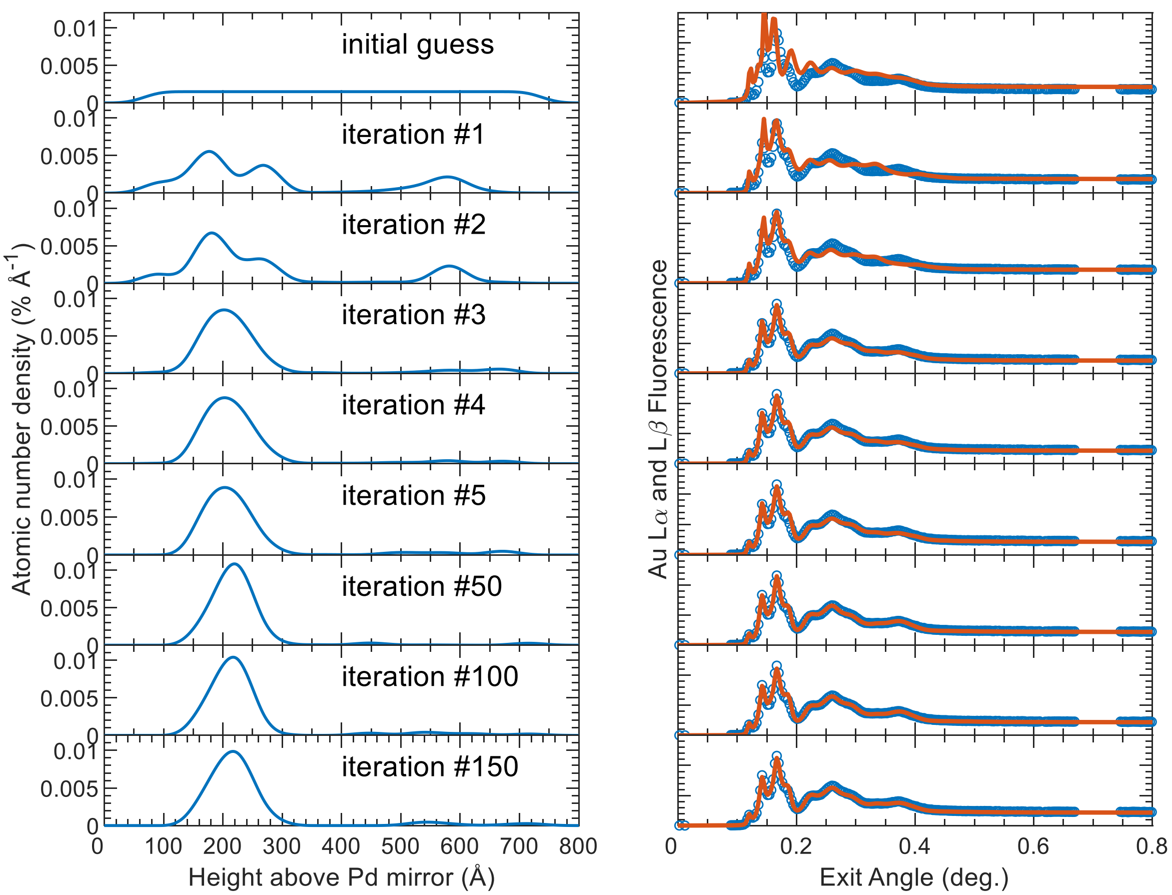

Supplementray Figure 3 Initial guess and selected HMCMC iterations for XWFH after annealing.

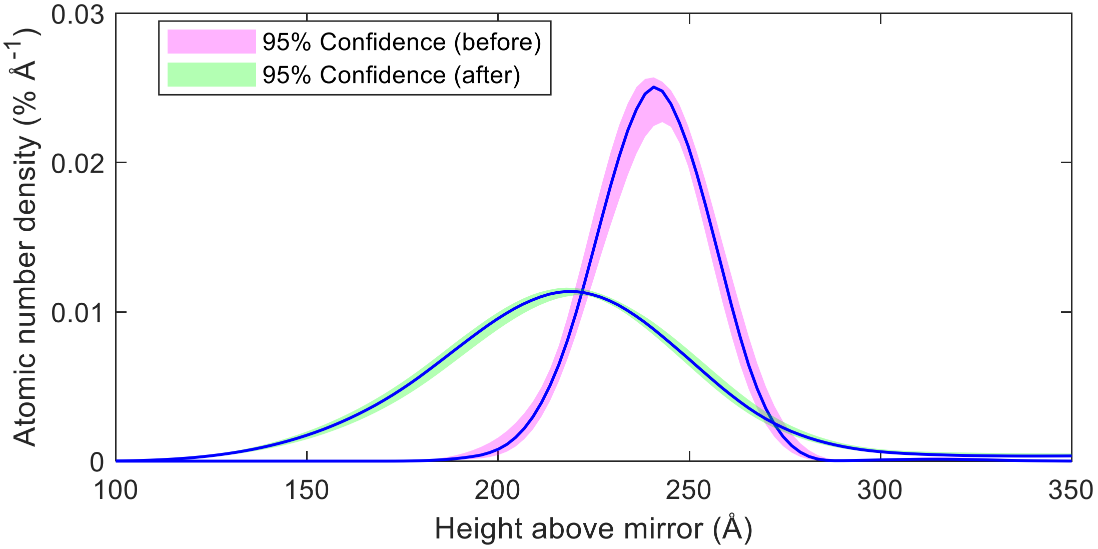

Supplementray Figure 4 95% confidence interval of the gold atom profile before and after thermal annealing. Sold lines correspond to the median profile (50% percentile).

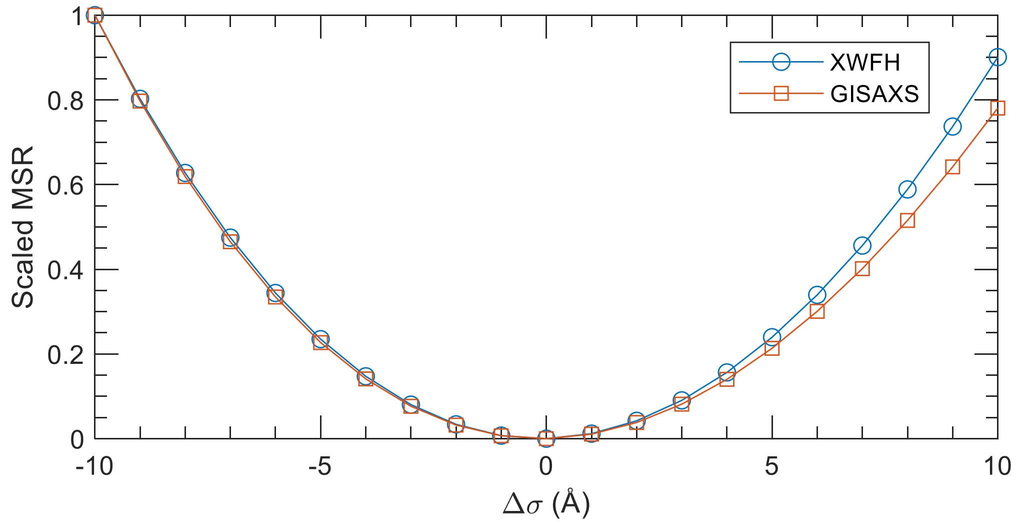

Supplementray Figure 5 Sensitivity comparison of XWFH and GISAXS using scaled MSR.

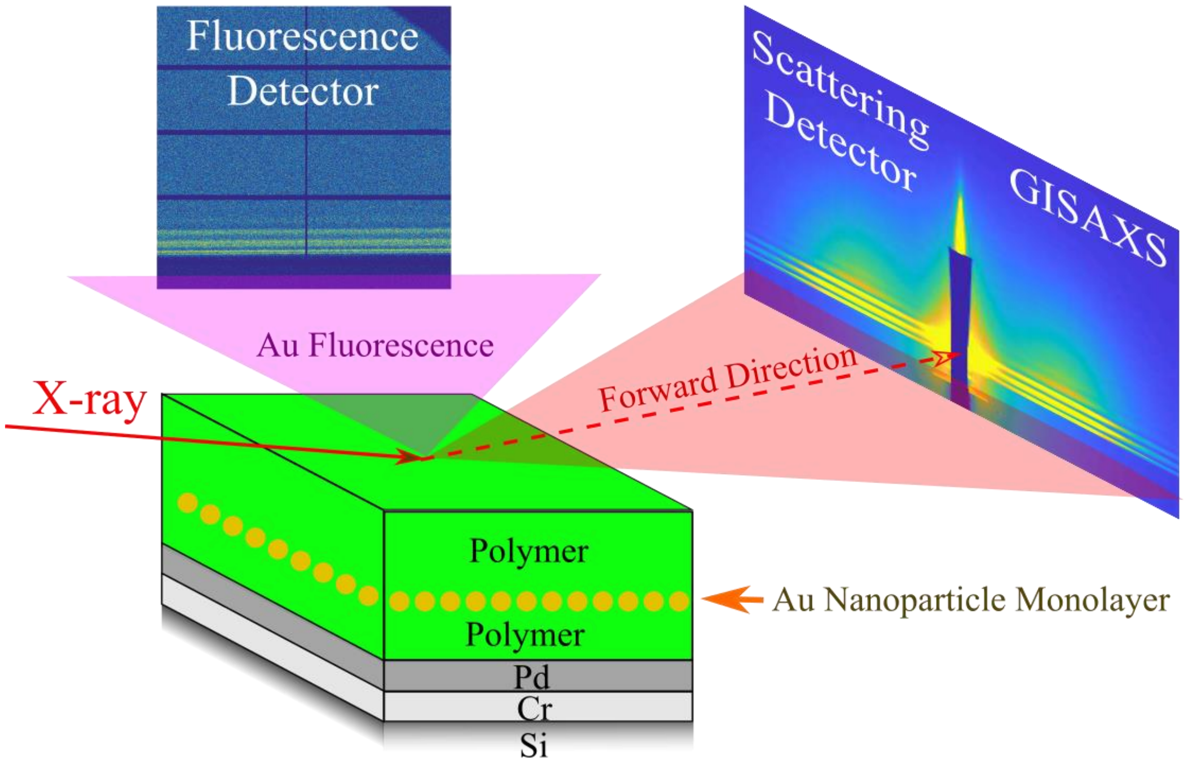

Supplementray Figure 6 Illustration of the experiment setup. GISAXS and reflectivity detector is mounted in the forward scattering direction, and the fluorescence detector is ~90 degrees in the horizon plane.

Supplementary Tables

Supplementray Table 1 Data for Figure 4e. Change of distribution variance due to out-of-plane diffusion of the gold monolayer for three samples measured with XWFH and GISAXS. The columns of each measurement are annealing time (sec), change of variance (Å^2), and its error bar (Å^2).

LL (XWFH)			HH (XWFH)			LH (XWFH)			LH (GISAXS)		
Time	Value	Error	Time	Value	Error	Time	Value	Error	Time	Value	Error
0	0	20.9	0	0	19.6	0	0	13.3	0	0	22.2
329	90.7	29.9	433	46.3	28	1787	360.1	23.6	1354	348.8	41.2
657	189.6	32.4	1305	183.2	30.5	2231	470.1	25.1	1787	376.3	32.2
986	260	33.1	1735	250.1	31.9	2665	543.1	26.2	2231	523.5	35.8
1314	324.1	33.4	2167	335	35.6	3089	617.5	27.8	2665	579	34.3
1643	391.1	34.9	2604	323.4	33.2	3526	712.2	30.3	3089	672.6	37.5
1971	475.9	40.8	3040	376.1	34.3	3950	778.9	31.2	3526	798	45.6
2300	625.8	48.4	3472	440.2	35	4383	903.6	34.7	3950	841.7	37.7
-	-	-	3904	468.7	37.2	4809	985.4	35.8	4383	980.2	48.1
-	-	-	4340	530.4	37.9	-	-	-	4809	1064.5	52.4

Supplementray Table 2 Data for Figure 4e inset. Fitted out-of-plane diffusion coefficients.

	Diffusion coefficient (Å^2/sec)	Error bar (Å^2/sec)
LL (XWFH)	0.2552	0.0141
HH (XWFH)	0.1237	0.0060
LH (XWFH)	0.2033	0.0130
LH (GISAXS)	0.2213	0.0056

Supplementray Table 3 Data for Figure 4f. Mean height change of the gold monolayer measured with XWFH. The columns are annealing time (sec), mean height (Å), and its error bar (Å).

LL (XWFH)			HH (XWFH)			LH (XWFH)		
Time	Value	Error	Time	Value	Error	Time	Value	Error
0	0	0.62	0	0	0.43	0	0	0.37
329	-3.39	0.86	433	-3.18	0.6	1787	-7.29	0.55
657	-5.17	0.89	1305	-3.59	0.63	2231	-7.74	0.55
986	-7.15	0.88	1735	-4.11	0.64	2665	-12.67	0.55
1314	-8.68	0.86	2167	-7.12	0.68	3089	-14.56	0.56
1643	-10.67	0.87	2604	-5.58	0.65	3526	-14.87	0.58
1971	-11.54	0.95	3040	-6.76	0.66	3950	-19.41	0.57
2300	-13.07	1.04	3472	-7.87	0.65	4383	-21.25	0.59
-	-	-	3904	-8.48	0.68	4809	-23.19	0.58
-	-	-	4340	-8.16	0.68	-	-	-

Supplementary References

1. Hastie, T., Tibshirani, R. & Friedman, J. *The Elements of Statistical Learning Data Mining, Inference, and Prediction (12th printing)*. (Springer New York, 2017).
2. Gamerman, D. & Lopes, H. F. *Markov Chain Monte Carlo: Stochastic Simulation for Bayesian Inference, Second Edition*. (Taylor & Francis, 2006).
3. Neal, R. M. in *Handbook of Markov Chain Monte Carlo* 113–162 (CRC Press, 2011).
4. Betancourt, M. A conceptual introduction to Hamiltonian Monte Carlo. Preprint at https://arxiv.org/abs/1701.02434v2 (2018).
5. Jiang, Z., Wang, J. Tirrell M. V., de Pablo, J. & Chen, W. Efficient inverse method for X-ray and neutron data with Subspace Hamiltonian Markov Chain Monte Carlo. Unpublished (2020).
